# 166. Congenital Syphilis in Minnesota, 2016-2020

**DOI:** 10.1093/ofid/ofab466.166

**Published:** 2021-12-04

**Authors:** Nicholas Lehnertz, Khalid Bo-Subait, Candy Hadsall, Cheryl Barber, Allison LaPointe, Cindy Lind Livingston, Karmen Dippmann, Marcie Babcock, Brian Kendrick, Jayne Griffith, Gina Liverseed, Peggy Darrett-Brewer, Christine L Jones

**Affiliations:** 1 Minnesota Department of Health, St. Paul, Minnesota; 2 MN Department of Health, St. Paul, Minnesota

## Abstract

**Background:**

Nationally, cases of congenital syphilis (CS) have increased over the past 5 years. We reviewed CS cases in Minnesota from 2016-2020.

**Methods:**

All cases of syphilis, including CS, are reported to the Minnesota Department of Health (MDH), including accompanying data on maternal age, baby’s sex, race, test results, maternal stage and treatment of mother and child. Medical records and case interviews were reviewed; the 2018 national case definition was used to classify cases.

**Results:**

During 2016-2020, there were 47 CS cases from 45 mothers, peaking in 2020 at a rate of 3.2/10,000 live births. 43 (91.5%) cases of CS had no clinical signs, 1 (2.1%) CS case was inadequately treated, and there were 2 deaths.

The median maternal age was 28 (IQR 9, range 18-38). 13 (28.9%) identified as Black, non-Hispanic, 13 (28.9%) as American Indian/Alaska Native (AI/AN), 9 (20.0%) as White, non-Hispanic, 3 (6.7%) as Hispanic, 2 (4.4%) as Asian/Pacific Islander, and 5 (11.1%) Other/Unknown. Twenty-four (51.1%) cases occurred in the Minneapolis/St. Paul metropolitan area. 2 (4.4%) cases were primary, 1 (2.2%) was secondary, while 18 (40.0%) maternal cases were staged as early non-primary, non-secondary (ENPNS) and 24 (53.3%) were late unknown duration. 14 (31.1%) of mothers had their initial prenatal visit in the first trimester, 6 (13.3%) in the 2^nd^ trimester, 11 (24.4%) in the 3^rd^, and 14 (31.1%) unknown. None of the maternal cases were HIV+, 2 were identified as positive for hepatitis C. 18 (40.0%) mothers had no or limited prenatal care, 21 (46.7%) had inadequate treatment for syphilis, and 18 (40.0%) had inadequate maternal testing. No cases reported substance use, but one case had a positive substance screen at delivery, and case interviews also documented a role of substance use and home instability in several other cases.

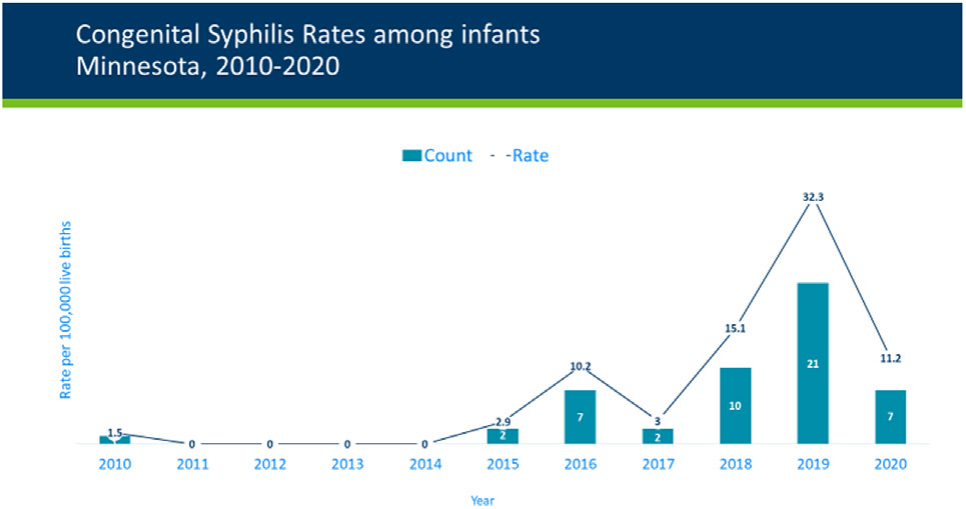

**Conclusion:**

Case rates of CS are the highest ever seen in MN. There is disproportionate impact in persons of color and indigenous Minnesotans. Lack of access to prenatal care, missed opportunities for testing, and incomplete or insufficient treatment were found in maternal cases. More work needs to be done with communities at risk and with prenatal care providers to ensure adequate testing, identification and treatment for syphilis in women of child-bearing age.

**Disclosures:**

**All Authors**: No reported disclosures

